# Biosynthesis and Characterization of Extracellular Silver Nanoparticles from *Streptomyces aizuneusis*: Antimicrobial, Anti Larval, and Anticancer Activities

**DOI:** 10.3390/molecules27010212

**Published:** 2021-12-30

**Authors:** Hemmat M. Abd-Elhady, Mona A. Ashor, Abdelkader Hazem, Fayez M. Saleh, Samy Selim, Nihal El Nahhas, Shams H. Abdel-Hafez, Samy Sayed, Enas A. Hassan

**Affiliations:** 1 Agricultural Microbiology Department, Faculty of Agriculture, Ain Shams University, Cairo 11566, Egypt; hemat_adbelhady@agr.asu.edu.eg (H.M.A.-E.); mona.ashor@agr.asu.edu.eg (M.A.A.); abdelkader_taha@agr.asu.edu.eg (A.H.); 2 Department of Medical Microbiology, Faculty of Medicine, University of Tabuk, Tabuk 71491, Saudi Arabia; fsaleh@ut.edu.sa; 3 Department of Clinical Laboratory Sciences, College of Applied Medical Sciences, Jouf University, Sakaka 72388, Saudi Arabia; sabdulsalam@ju.edu.sa; 4 Botany and Microbiology Department, Faculty of Science, Alexandria University, Alexandria 21515, Egypt; nihal.elnahhas@alexu.edu.eg; 5 Department of Chemistry, College of Science, Taif University, Taif 21944, Saudi Arabia; s.abdelhafez@tu.edu.sa; 6 Department of Science and Technology, University College-Ranyah, Taif University, Taif 21944, Saudi Arabia; s.sayed@tu.edu.sa

**Keywords:** microbial synthesis, AgNPs, identification, antibacterial, antifungal, biological activities, cytotoxicity, actinomycetes

## Abstract

The ability of microorganisms to reduce inorganic metals has launched an exciting eco-friendly approach towards developing green nanotechnology. Thus, the synthesis of metal nanoparticles through a biological approach is an important aspect of current nanotechnology. In this study, *Streptomyces aizuneusis* ATCC 14921 gave the small particle of silver nanoparticles (AgNPs) a size of 38.45 nm, with 1.342 optical density. AgNPs produced by *Streptomyces aizuneusis* were characterized by means of UV-VIS spectroscopy and transmission electron microscopy (TEM). The UV-Vis spectrum of the aqueous solution containing silver ion showed a peak between 410 to 430. Moreover, the majority of nanoparticles were found to be a spherical shape with variables between 11 to 42 nm, as seen under TEM. The purity of extracted AgNPs was investigated by energy dispersive X-ray analysis (EDXA), and the identification of the possible biomolecules responsible for the reduction of Ag^+^ ions by the cell filtrate was carried out by Fourier Transform Infrared spectrum (FTIR). High antimicrobial activities were observed by AgNPs at a low concentration of 0.01 ppm, however, no deleterious effect of AgNPs was observed on the development and occurrence of *Drosophila melanogaster* phenotype. The highest reduction in the viability of the human lung carcinoma and normal cells was attained at 0.2 AgNPs ppm.

## 1. Introduction

Microbial synthesis of nanoparticles can develop cost effective and eco-friendly methods to produce technologically important materials. Due to their chemical stability, biocompatibility, catalytic activity, high conductivity, and inherent medicinal properties, silver nanoparticles have a wide range of uses in a variety of industrial sectors, including medicine, pharmaceuticals, bimolecular detections, food production, agriculture, and the textile industry [1].

Silver nanoparticles can be synthesized by chemical, physical, and biological methods [2]. Several chemical methods have been developed for the synthesis of silver nanoparticles, including chemical reduction, aqueous solution, non-aqueous chemical reduction, the template method, electrochemical reduction, microwave-assisted synthesis, irradiation reduction, the microemulsion method, biochemical method, etc. Nonetheless, these chemical methods have been reported, along with various drawbacks, including the use of toxic solvents, generations of hazardous by-products, and high energy consumption, which pose potential risks to human health and to the environment [3,4].

Nanoparticles resulting from certain microbial processes have unique chemical and physical properties, different from properties conventionally known to nanoparticles, depending on the microorganisms and medium passed, as well as operational conditions applied [5].

Actinomycetes are considered an important resource for new products of medical and industrial interest, such as antimicrobial agents [6,7]. AgNPs produced by *sterptomyces albidoflavus* have a greater impact on industrial application spectra [8]. Mabrouk et al. [9] found that *Streptomyces spiralis* and *Streptomyces rochei* reduced silver nitrate into silver nanoparticles. The sizes of AgNPs produced were highly dependent on the amount of molecular size and diversity of extracellular matrix proteins of the microorganism. Furthermore, Salem et al. [10] recorded that *Streptomyces antimycoticus* was used as a biocatalyst for reducing, capping, and stabilizing AgNPs. The analysis of the biological activities of AgNPs produced revealed their efficacy as a bactericidal agent and their in-vitro cytotoxicity against a cancerous cells line.

The anticancer properties of silver nanoparticles against colon, brain, kidney, hepatobiliary, intestinal, and cervical cancer cell lines have recently been the subject of several studies [11]. Therefore, this study was carried out for *Streptomyces* AgNPs biosynthesis, as well as the detection of the characterization of AgNPs and their activities as an antimicrobial, antitumor, and toxogenic agent. 

## 2. Results

### 2.1. Biosynthesis of Silver Nanoparticles (AgNPs)

The synthesis of AgNPs by *Streptomyces aizuneusis* was carried out on a reaction mixture between culture supernatant and one mM silver nitrate solution. Therefore, there were two steps for production: biomass production in Malt extract Glucose Peptone Yeast extract medium (MGPY), and AgNPs synthesis. 

**Figure 1:**
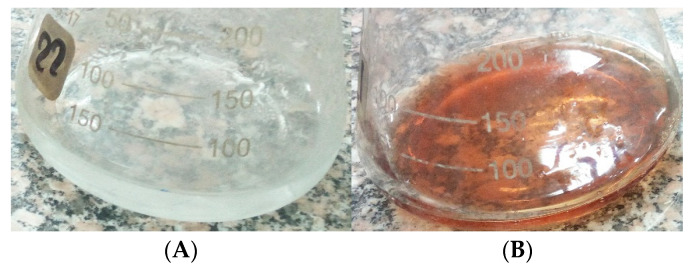
Biosynthesis of silver nanoparticles color change reaction in the present of extracellular filtrate of the *Streptomyces aizuneusis* ATCC 14921 biomass (**A**) and the presence of extracellular filtrate of the *Streptomyces aizuneusis* biomass exposure to AgNO_3_ solution after 7 days (**B**).

**Figure 2:**
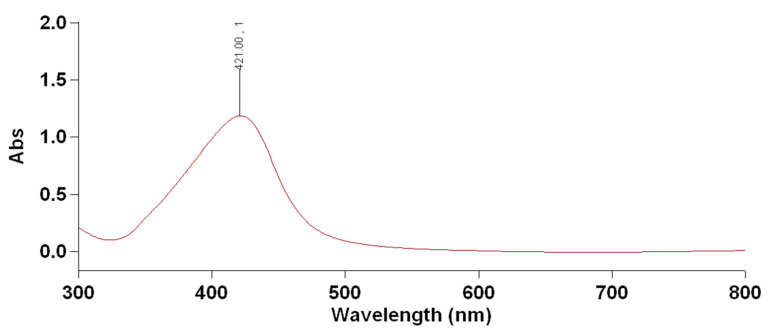
UV-Visible spectrum of silver nanoparticles produced by *Streptomyces aizuneusis* ATCC 14921.

*Streptomyces aizuneusis* ATCC 14921 strain recorded AgNPs particle size of 38.45 nm with the concentration of 1.342 as O.D. The color change from yellow (control A) to the brown color in the silver nitrate-containing flask treated by *Streptomyces aizuneusis* ATCC 14921 strain as a positive reaction (flask B), as illustrated in Figure 1. A UV-visible spectrum was obtained from 300–700 nm, while a clear peak at 400 nm was observed due to the presence of silver nanoparticles (Figure 2). Moreover, the silver nanoparticles solution was highly stable at room temperature for 8 weeks with no flocculation of particles, as determined by UV-vis spectroscopy measurements (unpublished data).

### 2.2. Characterization of Synthesized AgNPs

#### 2.2.1. UV-VIS Spectrum Analysis

Data illustrated in Figure 2 showed that a strong and broad peak was obtained between 410 nm and 430 nm, indicating the presence of AgNPs. Furthermore, as shown in Figure 2, the specific Surface Plasmon Resonance (SPR) confirmed the successful formation of AgNPs. Krishnaraj et al. [12] reported that the SPR patterns and characteristics of AgNPs were found to strongly depend on particle size, stabilizing molecules, or surface adsorbed particles, as well as the dielectric constant of the medium.

#### 2.2.2. Energy Dispersive X-ray Analysis (EDAX)

**Figure 3:**
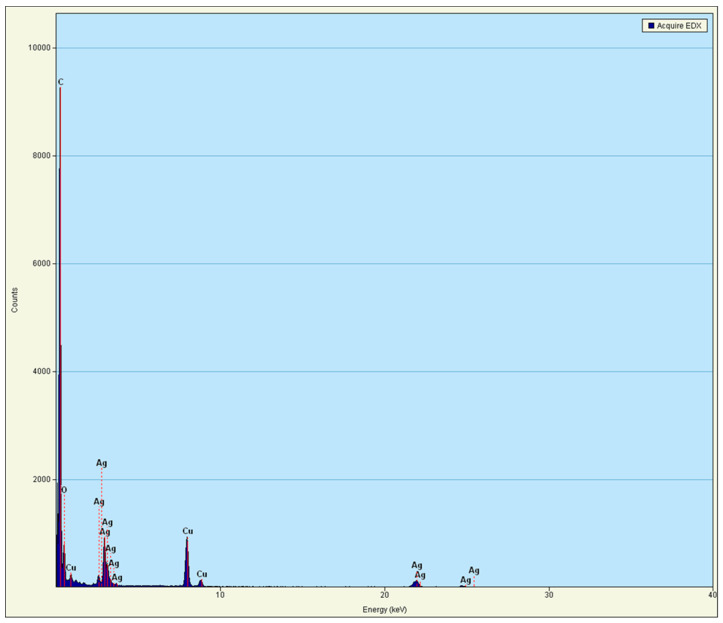
Energy Dispersive Absorption photograph (EDXA) of AgNPs produced by *Streptomyces aizuneusis* ATCC 14921.

The purity of the extracted AgNPs was investigated by EDXA (Figure 3). The EDXA spectrum showed a strong signal for silver at 3 Kev, which is typical for metallic silver nano-crystallites, according to Kohler et al. [13]. The other peaks were also recorded (cu), most likely due to the borosilicate glass on which the sample was coated (from the grid used) [14,15].

#### 2.2.3. TEM Analysis

**Figure 4:**
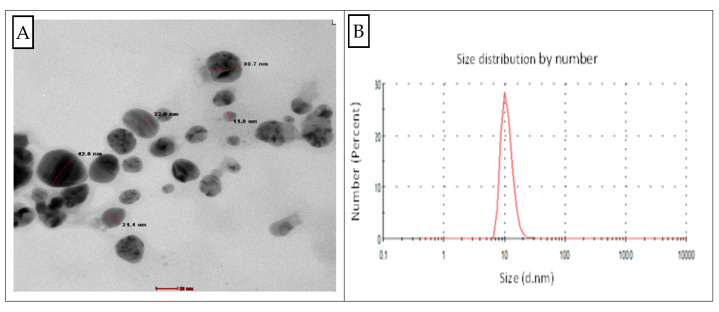
Transmission electron microscopy (TEM) micrograph of AgNPs produced by *Streptomyces aizuneusis* (**A**) and particle size distribution curve (**B**).

The TEM micrograph provided the shape and particle size of AgNPs biosynthesis by *Streptomyces aizuneusis* ATCC 14921. The TEM image in Figure 4 indicated the spherical-shaped AgNPs as well as their diameter, which was between 11 nm and 42 nm. The result referred that this particle was polydispersed with variable diameters ranging from 11 to 42 nm.

#### 2.2.4. Fourier Transform Infrared Spectrum (FTIR) 

The spectra of silver nanoparticles based on vibrational assignments/functional groups corresponding to absorption peaks were analyzed by Fourier Transform Infrared spectrum (FTIR). FTIR spectrum showed a peak at 647.06, 1172.2, 1382.08, 1634.45, 2078.78, 2808.52, 2880.65, and 3452.89 cm^−1^, respectively (Figure S1). The stronger band observed at 647.06 corresponded to C-S stretching of chloro-alkanes; the band appeared at 1382.08, referring to C-C stretching aromatics, while the bands observed at 2078.78 and 2880.65 were assigned to aromatic-CH stretching and methyne C-H stretch, respectively. The broadband observed at 3452.89 cm^−1^ could be assigned to stretching vibrations of O-H group. The stronger band found at 1634.45 cm^−1^ could be assigned to the characteristic N-H bend of primary amine.

#### 2.2.5. Zeta Potential Analysis

The stability of biosynthesized silver nanoparticles depended on both size and surface charge of the nanoparticles. The data obtained from Dynamic Light Scattering (DLS) measurements indicated that the size of the particles were varied, and ranged from 11–42 nm, with a negative charge of −26 ± 0.2 mV (Figure S2). The high negative value supported long-term stability, good colloidal nature, and high dispersity of biosynthesized silver nanoparticles due to negative repulsion. Tashi et al. [16] reported that a minimum zeta potential was required for the indication of stable AgNPs.

#### 2.2.6. X-ray Diffraction (XRD)

XRD analysis showed two distinct diffraction peaks at 38.15° and 44.51° for cubic face-centered silver. The lattice constant calculated from this pattern was: a = 3.36 °A (Figure S3).

#### 2.2.7. Antimicrobial Activity of AgNPs

Data presented in Table 1 revealed that all silver nanoparticles concentration ranged from 0.01 to 0.07 ppm and inhibited the growth of all tested bacteria (Gram positive and Gram negative) and fungi, except *Salmonella paratyphi,* which was not affected higher than 0.02 ppm. The largest inhibition zone diameter was recorded against *Pseudomonas aeruginosa* (being 22 mm), followed by *Staphylococcus aureus* (being 20 mm), at AgNPs concentration of 0.07 and 0.02 ppm, respectively. At 0.07 ppm AgNPs, both fungal strains *sclerotium rolfsii* and *fusarium solani* gave the same susceptibility for 0.07 ppm AgNPs and recorded the largest inhibition zone diameter of 14 mm. Increasing the AgNPs concentration from 0.01 to 0.07 ppm led to an increase in their inhibition effect of about 25–30% for all tested strains. Therefore, a concentration of AgNPs 0.07 ppm exhibited the highest growth suppression against all tested strains. Based on these results, it can be concluded that the AgNPs biosynthesis had good antibacterial and antifungal actions at a low concentration.

#### 2.2.8. Toxicity of Silver Nanoparticles against *Drosophila melanogaster* Larvae

Data in Table 2 showed the percentages of normal flies and abnormal flies after 14 days of larval feed on soluble AgNPs using different concentrations individually. The AgNPs had no deleterious effect on development and occurrence of distinct phenotypes. The larvae which were fed on 0.01 ppm and 0.05 ppm gave 100% and 99.3% of the normal adult flies, respectively. Alternatively, the highest concentration of AgNPs (0.07 ppm) gave 98.8% of normal adult flies. This meant that no toxic effect of AgNPs led to gene mutation during larval development even with increasing the applied AgNPs concentrations.

#### 2.2.9. Silver Nanoparticles as Antitumor

**Figure 5:**
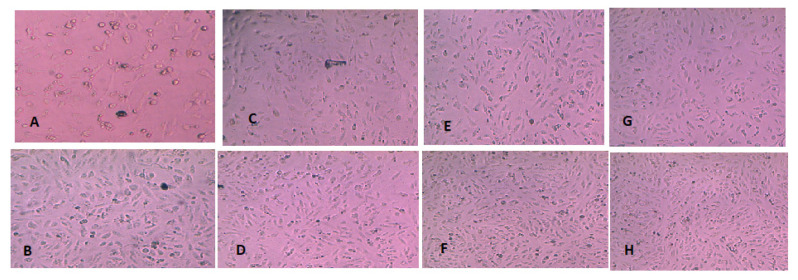
The cytotoxicity of different AgNPs concentrations produced by *Streptomyces aizuneusis* against liver carcinoma cell line detected by NRU colorimetric technique. The images were taken using microscopy at 100×. (**A**) negative control, (**B**) positive control, (**C**) cells treated with 0.01 ppm, (**D**) cells treated with 0.02 ppm, (**E**) cells treated with 0.04 ppm, (**F**) cells treated with 0.06 ppm, and (**G**) cells treated with 0.08 ppm and (**H**) cells treated with 0.1 ppm.

Different concentrations of AgNPs produced by *Streptomyces aizuneusis* ATCC 14921 (0.01, 0.02, 0.04, 0.06, 0.08, and 0.1 ppm) were added on human liver carcinoma (Hep2) cell line to determine their cytotoxicity, as illustrated in Figure 5. They gradually changed in the color of solution from yellow to brown and a reduction in the cell viability was followed. All treated tumor cells were inhibited with all the tested concentrations, beginning from 0.01 to 0.1 ppm, giving a zero percentage of viability of cells, compared to negative control (100% viability for non-treated liver cells) and positive control (100% cell death when treated by cisplatin as an anticancer chemical component).

Furthermore, results in Table 3 showed the cell viability of treated normal or cancer human lung cell by different concentrations of silver nanoparticles produced by *Streptomyces aizuneusis* ATCC 14921. The effect of AgNPs on cells exhibited a dose-dependent toxicity. At 0.02 ppm of AgNPs of *Streptomyces aizuneusis* ATCC 14921, the cell viability of cancer cells was 45.4%, whereas against normal cells it was 89.6%. The viable cell count decreased gradually with increasing *Streptomyces aizuneusis* AgNPs concentration giving 4.6% viability at 0.2 ppm with cancer cells. The half maximum inhibiting concentration was 0.06 mg and 0.0148 ppm for normal and cancer cells, respectively. 

**Figure 6:**
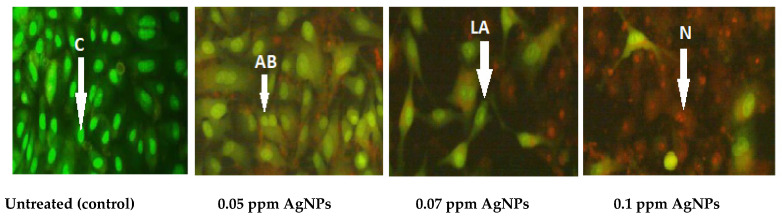
Apoptosis of lung cancer cell line (A549) after treated with AgNPs produced by *Streptomyces aizuneusis* ATCC 14921. The images were taken using fluorescence microscopy at 20×. C: control cells exhibited green normal nucleus; AB: early apoptosis cells showed bright green nucleus with fragmented chromatin; LA: late apoptosis cells showed condensed red nucleus; AB: an apoptotic body; N: necrosis.

Silver nanoparticle treated cells showed apoptotic morphological changes, as illustrated by Figure 6. Apoptotic morphological changes caused by silver nanoparticles were studied using acridine orange/ethidium bromide differential staining method. The stained cells were characterized to viable (light green), early apoptotic (bright green fluorescence and condensed chromatin), late apoptotic (orange fluorescence), and nonviable cells (red-colored fluorescence). Silver nanoparticle treated cells showed condensed nuclei, membrane blebbing, and apoptotic bodies. In contrast, the control cells showed intact nuclear architecture. 

## 3. Discussion

The actinobacteria recorded four distinct proteins of molecular masses between 10 and 80 kDa. One or more of these proteins may have been enzymes that reduced the metal ion by the reduction process. It was also possible that the capping and stabilization of silver nanoparticles was affected by a different protein [17]. Due to their quick growth and high biomass, actinomycetes, such as Streptomyces, were ideal candidates for large-scale biological manufacture of nano-sized materials; they’re also known for their cost-effectiveness and environmental friendliness [18], and even more interestingly was the fact that actinobacteria produced enzymes and proteins that might be used in nanoparticle manufacturing [19]. 

In this work, *Streptomyces aizuneusis* ATCC 14921 gave the AgNPs particles a size of 38.45 nm with a concentration of 1.342. In addition, washed actinomycetal biomass filtrate was treated with an aqueous solution of AgNO_3_, which led to the appearance of color change, indicating the reduction of the metal ions and formation of silver nanoparticles, since AgNPs exhibit striking colors (light yellow to brown), due to excitation of surface Plasmon vibration in the particle [20]. The concentration of AgNPs produced by this strain was also determined by Atomic Absorption Spectrophotometer to be 1632 mg/L. Soliman et al. [21] determined the color intensity (in most cases) was a direct result of the number of reduced Ag ions, which in turn correlated with the reduction efficacy.

The investigation AgNPs scan were determined by UV spectrophotometer. It was observed that all the spectra prosses main band around 403, which indicated the formation of AgNPs early reported [22,23]. The bioreduction of silver metal ions may have occurred due to the presence of secondary cell metabolites [15]. The SPR for AgNPs produced by *Streptomyces aizuneusis* ATCC 14921 did not vary over different intervals of time, confirming the long-term stability of the biosynthesized AgNP (data unpublished). The AgNPs prepared by tested culture showed spherical nanoparticles with a diameter range of 11–42 nm, where the nanoparticles were monodispersed without any aggregation. Hamouda et al. [24] found that silver nanoparticles could be stabilised and reduced using cyanobacterial water extracts, which were spherical monodispersed silver nanoparticles.

The XRD pattern revealed peaks at 38.15 and 44.5. The recorded peaks of AgNPs were in accordance with XRD pattern observed by Khatami et al. [25]. In agreement with our results, Abd-Elnaby et al. [26] reported the extracellular synthesis of silver nanoparticles from *Streptomyces rochei* with the particle size range of 22–85 nm. The FTIR spectrum identified the possible biomolecules responsible for the reduction of Ag^+^ ions by the cell filtrate. Most bands found in the produced AgNPs are similar to the bands obtained by Hemath Naveen et al. [27]; Yokesh Babu et al. [28], and Sanjivkumar et al. [15]. 

The peaks in the infrared (IR) spectrum could be attributed to the presence of functional groups (e.g., C-N, -OH, C=O, C-C), which may have formed due to the interactions of metabolites (e.g., proteins) with the biofabricated AgNPs. These results were consistent with those obtained by El-Naggar et al. [29] and Saravanan et al. [30], who reported that the functional group (or peaks) such as C=O and C-C arise from heterocyclic compounds such as proteins, which acted as capping agents for NPs. The peaks EDXA spectrum recorded a strong signal for silver at 3 Kev and most likely (cu) due to the cover of the sample.

The value for Ag-NPs was recorded as −26 ± 0.2 mV, which reflected the stability of the biosynthesized NPs solution. In similar studies by Alsharif et al. [31] and Elamawi et al. [32], the values of AgNPs synthesized by *Streptomyces noursei* H1–1 and *Trichoderma longibachiatum* were −18.9 mV and 19.7 mV, respectively. 

Undoubtedly, the antibacterial activity of AgNPs was expected to possess different mechanisms compared to antibiotics [33]. Generally, the antibacterial property of AgNPs was essential due to the delivery of silver cations from AgNPs that worked as a source for these nanoparticles. Pallavicini et al. [34] focused on the preparation of a monolayer of silver nanoparticles (AgNP) and their use as antimicrobial surfaces against both planktonic bacteria and biofilms, as well as the role of Ag^+^ release and of direct cell/AgNP contact in the antibacterial action as a function of the adhesive molecular layer, of the AgNP dimension and shape, of their surface density, and of the molecular overcoating [34]. In this study, the tested bacterial strains were more sensitive than fungal strains at different AgNPs concentrations. All different concentrations of AgNPs tested significantly affected all tested microorganisms, but no significant effect was observed by 0.02 and 0.05 ppm for *Streptomyces aizuneusis* AgNPs on bacterial strains and by 0.01 and 0.02 ppm on the growth of fungal strains. No significant differences of AgNPs at different concentrations were observed against *Staphylococcus aureus* and *P. aeruginosa*. Increasing the AgNPs concentration from 0.01 to 0.07 ppm led to an increase in their inhibition effect of about 25–30% for all tested strains. As such, the concentration of AgNPs 0.07 ppm exhibited the highest growth suppression against all tested strains. Based on these results, it can be concluded that the AgNPs biosynthesis had good antibacterial and antifungal actions at low concentrations. These results agree with Avilala and Golla [35] who reported that the biosynthesized AgNPs produced by *Streptomyces hygroscopicus* inhibited the medically important pathogenic bacteria (*Bacillus subtilis, Enterococcus faecalis, Escherichia coli,* and *Salmonella typhimurium*). In the context, Wypij et al. [36] reported the biogenic synthesis of AgNPs from *Streptomyces xinghaiensis* OFI with efficient nano-antibiotic properties against *B. subtilis*, *S. aureus, E. coli*, and *P. aeruginosa*. 

The rapid development of nanotechnology allowed the fabrication of a wide range of different nanomaterials, raising many questions about their safety and potential risks for human health and the environment. *Drosophila melanogaster* provided a powerful model for investigating human health and nanotoxicity of nanoparticles. The *D. melanogaster* model offered several important advantages, such as a relatively simple genome structure, short lifespan, low maintenance cost, readiness of experimental manipulation comparative to vertebrate models from both ethical and technical points of view, relevant gene homology with higher organisms, and ease of obtaining mutant phenotypes. The great similarities between humans and flies were recognized and appreciated by scientists working in biology and medicine, making *Drosophila* the non-mammal model organism par excellence, even in those disciplines where mammal model organisms were considered irreplaceable (i.e., pharmacology [37,38] and genotoxicology) [39]. In this experiment, laboratory trials were conducted to determine the toxicity effectiveness of AgNPs against *Drosophila* larval development. Different concentrations of *Streptomyces aizuneusis* AgNPs (0.01, 0.05, and 0.07) had no deleterious effects on the development and occurrence of *Drosophila melanogaster* larvae.

These results were in agreement with Araj et al. [40], who reported that none of the tested silver nanoparticles had a significant effect on pupae longevity of *Drosophila.* On the contrary, Tyagi [41] reported that silver nanoparticles ingestion during the larval stage resulted in cuticular and melanization defects in adults. Flies that survived higher concentrations of AgNPs ingestion had soft, non-pigmented cuticles. No such effect was observed in the normal-fed flies as control; where the control flies had higher pigmented in all abdominal segments compared to AgNPs feds flies in both sexes. Furthermore, Araj et al. [40] reported that silver nanoparticles (AgNPs) were highly effective on larvae, pupa, and adult’s mortality and egg deterrence. Therefore, it was imperative that more studies be carried out to determine the toxicity effect of nanosilver.

The literature proved that nanosilver could induce a toxic effect on proliferation and cytokine expression by peripheral blood mononuclear cells [42,43]. The neutral red uptake assay (NRU) procedure was a cell survival/viability assay, based on the ability of viable cells to incorporate and bind neutral red within lysosomes. Therefore, it could be used to evaluate the cytotoxicity of bio-silver nanoparticles by determining the IC50 (50% inhibiting concentration). The NRU cell survival assay was widely used for measuring the cytotoxic potential of a compound. Cytotoxicity was expressed as a concentration-dependent reduction of the uptake of NR after chemical exposure and serves as a sensitive indicator of both cell integrity and growth inhibition. 

In the present study, the efficacy of biological synthesized AgNPs as an antitumor agent was determined on human lung and liver carcinoma cell line in vitro by NRU colorimetric techniques. The toxicity effect showed a reduction in cell count in tumor cells treated with AgNPs. All treated human liver carcinoma were inhibited by different concentrations of AgNPs (0.01 to 1 ppm), whereas the viable cell count decreased gradually with increasing AgNPs concentration giving 4.6% viability at 0.2 ppm, and the IC50 were 0.06 and 0.0148 ppm for a normal and human lung cancer cell, respectively. A similar study was reported by Sriram et al. [44], who demonstrated the efficacy of biologically synthesized AgNPs as an antitumor agent using Dalton’s lymphoma ascites (DLA) cell lines in vitro and in vivo. They added the histopathologic analysis of ascitic fluid showing a reduction in DLA cell count in tumor-bearing mice treated with AgNPs. Further, Kaler et al. [45] reported that silver nanoparticles at very low concentrations showed very high activity on MCF-7 cells, showing almost 80% inhibitions. At higher concentrations (10–100 mg/mL) no significant difference in inhibition of cancer cells was observed with silver nanoparticles, and the IC50 value for the silver nanoparticles was less than 10 mg/mL. Meanwhile, Shawkey et al. [46] and Salem et al. [10] found that the HCT-116 and Hep-G2 cell lines were the most sensitive cell lines towards the cytotoxic activity of AgNPs, while the Caco-2 cell line was the most resistant cell line towards cytotoxic activity.

## 4. Materials and Methods

### 4.1. Microorganisms Used

The actinomycetes *Streptomyces aizuneusis* ATCC 14921, bacterial strains used for antimicrobial detection (*Pseudomonas aeruginosa*, *Staphylococcus aureus*, *Bacillus subtilis*, *Listeria monocytogenes*, *Salmonella paratyphi*, and *Escherichia coli* O157:H7) were obtained from the Department of Agriculture Microbiology, Ain Shams University, Faculty of Agriculture, Cairo, Egypt. Phytopathogenic fungal strains (*Fusarium solani*, *Sclerotium rolfsii*, *Ralistonia solani*) were obtained from Agricultural Research Center, Giza, Egypt.

### 4.2. Media Used

Malt extract Glucose Peptone Yeast extract medium (MGPY) [17] was used to prepare the biomass for biosynthesis studies. Mueller Hinton agar medium [47] was used to determine the antimicrobial activities of AgNPs. It typically contained (g/L): beef extract, 2; casein hydrolysate, 17.5; starch, 1.5; agar, 20.

### 4.3. Standard Inoculum

Standard inoculum of the tested strain was prepared by inoculation the plates containing Glycerol-nitrate agar medium with a loop of tested culture, then incubated at 30 °C for 72 h. 

### 4.4. Biosynthesis of AgNPs

The synthesis of AgNPs by *Streptomyces aizuneusis* ATCC 14921 in all experiments was carried out on a reaction mixture between the culture filtrate and 1 mM silver nitrate solution. Therefore, there were two steps for production: biomass production and AgNPs synthesis, as follows. 

The propagation of *Streptomyces aizuneusis* ATCC 14921 was carried out in Erlenmeyer flasks (250 mL in volume) containing 50 mL MGPY medium. These flasks were inoculated with one fresh disk of actinomycetes culture (7 mm in diameter). The inoculated flasks were incubated at 30 °C for 96 h on an orbital shaker (150 rpm). At the end of the propagation period, the biomass was harvested by centrifugation at 6000 rpm for 20 min, followed by extensive washing with distilled water to remove any medium component from the biomass. Freshly collected biomass was added in 50 mL of sterile distilled water for 48 h at 30 °C in an Erlenmeyer flask and agitated at 150 rpm to release the intracellular metabolites, which were obtained after the final filtration for AgNPs synthesis. After the incubation, the cell filtrate was obtained carefully, by passing it through Whatman filter paper no. 1. The resultant cell filtrate (50 mL) was then mixed with 50 mL of fresh AgNO_3_ solution (1 mM) and kept on a shaker (150 rpm) at 30 °C under dark conditions for 7 days [48].

### 4.5. Characterization of Biosynthesized AgNPs

#### 4.5.1. UV-Visible Spectral Analysis

The bioreduction of silver ions of the reaction solutions (AgNO_3_ + cell filtrate) was observed by changes in color after the incubation period using a UV-Visible Spectrophotometer (CCT-2200) from 200–800 nm at regular intervals [49].

#### 4.5.2. Transmission Electron Microscopic (TEM)

Transmission electron microscopy (TEM) was carried out to investigate AgNPs morphological shape and diameter. For the analysis, a drop of a liquid solution containing AgNPs was placed on carbon-coated copper grids and allowing the water to evaporate inside a vacuum dryer [21,50].

#### 4.5.3. FTIR Spectroscopy Analysis

The synthesis AgNPs by the most potent culture was subjected to FTIR spectroscopy. Examined samples were mixed with 200 mg KBr (FTIR-grade) and pressed into a pellet, then placed into the sample holder. The FTIR spectrum was recorded in range 400–4000 cm^−1^ at a resolution of 4 cm^−1^.

#### 4.5.4. Energy Dispersive X-ray (EDXA) Analysis

EDXA were used to determine the elements contained in the extracted AgNPs sample produced by the most potent bacterial culture.

#### 4.5.5. Particle Size and Zeta Potential Analysis

Zeta potential was studied to determine the surface potential of AgNPs, since this character was essential for its stability in aqueous silver nanoparticles. The particle size and its zeta potential of biosynthesized silver nanoparticles by the most efficient isolates were determined using particle size analyzer (zeta sizer Nano-series, Malvern, UK) located at Egyptian Petroleum Research Institute.

#### 4.5.6. X-ray Diffraction

The X-ray crystallography was a technique used for determining the atomic and molecular structure of a crystal (crystalline nature), in which the crystalline atoms caused a beam of incident X-rays to diffract into many specific directions. This technique was performed at Egyptian Petroleum Research Institute.

### 4.6. Some Activities of Biosynthesized Silver Nanoparticles

#### 4.6.1. Antimicrobial Activity of AgNPs

The antimicrobial activity of AgNPs (produced by the *Streptomyces aizuneusis*) was measured against different pathogenic organisms; using a well diffusion method. Activated pure culture of tested bacteria was inoculated in sterile Mueller Hinton agar medium individually, and was then poured in sterile Petri dishes and allowed to solidify. Wells (6 mm in diameter) were made on these dishes and the biosynthesized AgNP was added with different concentrations ranging from 0.01, 0.02, 0.05, and 0.07 ppm individually into each well and incubated at 37 °C for 2 days. After incubation, the diameter of the inhibition zone around the well was estimated [51].

#### 4.6.2. Silver Nanoparticles Toxicity against *Drosophila melanogaster* Larvae

Rearing *Drosophila melanogaster*: *D. melanogaster* adults were obtained from an available colony at the Drosophila Laboratory at Genetics Department, Faculty of Agriculture, Ain shams University, Cairo, Egypt. These insects were grown into new culture bottles to breed. The composition of *Drosophila* artificial diet has the following composition (g/L): 48 sucrose, 18 bacteriological agar, 18 yeast, 4 mL propionic acid, and 54 wheat cream, Distilled water up to 1000 mL. Fruit flies were fed at the larvae stage on soluble silver nanoparticles produced by microbial culture to assess their toxicity by recording the number of normal or abnormal adult insects.

Bioassay test: Different concentrations of silver nanoparticles produced (i.e., 0.01 ppm, 0.05 ppm, and 0.07 ppm) were examined against *Drosophila melanogaster*. The AgNPs were mixed with the artificial diet in bottles individually. Fifty freshly hatched *Drosophila* larvae were transferred to each bottle with or without the nanoparticle impregnated in the diet. Each treatment was repeated three times. The artificial diet was treated with distilled water as a control treatment. The bottles were incubated at 25 °C, 60% relative humidity, and 16:8 lights to dark period for 24 h. After that, the larvae were transported to another bottle without any AgNPs to observe the larvae mortality. In addition, pupal mortality was recorded for those larvae that succeeded in pupation. Larval and pupal development periods were checked every 24 h and recorded. The larvae that succeeded in pupation were numbered on the outside surface of the bottles opposite to the pupation site with a permanent marker in order to follow its development period. Moreover, abnormalities in morphology and color of the emerged flies were noticed and recorded as a percentage effect [52].

#### 4.6.3. Antitumor Activity

This experiment was performed by neutral red assay according to cytotoxicity protocol provided by Repetto et al. [53]. This technique was used to detect the efficiency of silver nanoparticles produced by tested culture against normal or cancer lung and liver cell lines using different concentrations of silver nanoparticles. The viability of treated cells (%) of normal and cancer cell line was calculated for having the concentration of the test substance reflecting the half-maximum inhibitory concentration of the cell proliferation (IC50). Furthermore, an apoptosis assay was performed. This cytotoxicity assay was assessed in the cell culture lab, Research Park, Faculty of Agriculture, Cairo University.

Apoptosis assay: was carried out according to cytotoxicity protocol provided by Jennie et al. [54]. The cell suspension was counted using a hemocytometer, and cell viability was checked by trypan blue. The plate was examined under an inverted fluorescent microscope (LEICA DMI3000 B) filter I3, recording changes in morphology and color changes of the nucleus due to cytotoxic effects of each concentration.

## 5. Conclusions

This study was performed to produce biologically synthesized AgNPs using *Streptomyces aizuneusis* ATCC 14921, which was characterized with its size being 38.45 nm by particle size analyzer. TEM analysis showed that the particles were spherical in shape. Furthermore, the biosynthesized AgNPs had good antibacterial and antifungal action against all pathogenic tested strains at low concentrations. In other applications, no toxic effect of AgNPs produced was observed on *Drosophila* larval phenotypes after 14 days feeding period. Low concentration (0.01 ppm) of AgNPs produced showed very high inhibition activity for human lung and liver carcinoma cells. All treated liver tumor cells were inhibited with concentrations from 0.01 to 0.2 ppm. The half-maximum inhibiting concentration of *Streptomyces aizuneusis* ATCC 14921 for human lung carcinoma cells (IC50) was 0.0148 ppm.

## Figures and Tables

**Table 1:** Antimicrobial activity of different AgNPs concentrations produced by *Streptomyces aizuneusis* ATCC 14921 against some pathogenic microorganisms.

Pathogenic Microorganisms		Zone of Inhibition (mm)				Mean
		Concentration of AgNPs (ppm)				
		0.01	0.02	0.05	0.07	
Bacteria	*Staphylococcus aureus* *E. coli*	19	20	20	20	20.03 A
		11	12	13	14	13.03 C
	*Pseudomonas aeruginosa*	17	20	21	22	20.25 A
	*Listeria monocytogense*	14	16	16	17	16.5 B
	*Salmonella paratyphi*	0	12	14	16	10.81 D
	*Bacillus subtilis*	11	12	14	15	13.25 C
Mean		12.44 C	15.9 B	16.5 B	17.8 A	

Fungi	*Sclerotium rolfsii*	11	12	12	14	12.12 A
	*Rhizoctonia solani*	10	11	12	13	11.50 AB
	*Fusarium solani*	10	11	11	14	11.30 B
Mean		10.33 C	11.00 C	11.8 B	3.50 A	

Values in the same column followed by the same letter do not significantly differ from the other, according to Duncan, 1955 at 5% level.

**Table 2:** Toxic effect of silver nanoparticles at different concentrations on development stage of *Drosophila melanogaster*.

Silver Nanoparticles Concentrations (ppm)	Percentage of Normal Fly	Percentage of Abnormal Fly
0.01	100 ± 0.01	0 ± 0.01
0.05	99.3 ± 0.003	0.69 ± 0.003
0.07	98.85 ± 0.004	1.22 ± 0.004

All values were expressed as mean ± SE. Difference from the treatments at *p* < 0.05.

**Table 3:** The percentage of survival lung cells treated by different concentrations of AgNPs produced by *Streptomyces aizuneusis* cell filtrate.

Silver Nanoparticles Concentration (ppm)	Viability %	
	Normal Cell (Wi38)	Cancer Cell (A549)
0.02	89.6 ± 0.21	45.4 ± 0.14
0.05	71 ± 0.70	7.6 ± 0.21
0.07	22.3 ± 0.11	6.5 ± 0.17
0.1	9 ± 1.1	5.8 ± 0.28
0.2	3.3 ± 0.11	4.6 ± 0.21
The half maximal inhibitory conc. (IC50)	0.06	0.0148

Values are expressed as mean ± SE.

## Data Availability

Not applicable.

## Supplementary Materials

The following supporting information can be downloaded, Figure S1: Fourier Transform Infrared spectrum (FTIR) of AgNPs produced by *Streptomyces aizuneusis* ATCC 14921; Figure S2: Zeta potential of silver nanoparticles produced by *Streptomyces aizuneusis* ATCC 14921 using zeta sizer analyzer; Figure S3: X-ray diffraction of silver nanoparticles produced by *Streptomyces aizuneusis* ATCC 14921. Position (°2ϴ) (copper (cu)).

## Sample Availability

Samples of the compounds are not available from the authors.
