# Supplementary material for: Biosynthesis and Characterization of Extracellular Silver Nanoparticles from Streptomyces aizuneusis: Antimicrobial, Anti Larval, and Anticancer Activities
Source: Molecules. 2021 Dec 30;27(1):212. doi: 10.3390/molecules27010212 (PMC8746530; doi:10.3390/molecules27010212)
Supplement: Supplementary file 1 [file molecules-27-00212-s001.zip › molecules-1484406-supplementary.pdf]

molecules

Biosynthesis, Characterization of Extracellular Silver Nanoparticles from *Streptomyces aizuneusis*: Antimicrobial, Anti Larval and Anticancer Activities

Hemmat M. Abd-Elhady [1], Mona A. Ashour [1], Abdelkader Hazem [1], Fayez M Saleh [2], Samy Selim [3], Nihal El Nahhas [4] Shams H. Abdel-Hafez [5], Samy Sayed [6] and Enas A. Hassan [1*]

1. Faculty of Agriculture, Ain Shams University, Agricultural Microbiology Department, Cairo, Egypt; hemat_adbelhady@agr.asu.edu.eg (H.A); mona.ashor@agr.asu.edu.eg (M.A); abdelkader_taha@agr.asu.edu.eg (A.H); enas_elsayed@agr.asu.edu.eg (E.H)
2. Department of Medical Microbiology, Faculty of Medicine, University of Tabuk, Tabuk 71491, Saudi Arabia; fsaleh@ut.edu.sa (F.S)
3. Department of Clinical Laboratory Sciences, College of Applied Medical Sciences, Jouf University, Sa-kaka 2014, Saudi Arabia; sabdulsalam@ju.edu.sa
4. Botany and Microbiology Department, Faculty of Science, Alexandria University, Alexandria 21515, Egypt; nihal.elnahhas@alexu.edu.eg (N.N)
5. Department of chemistry, College of Science, Taif University, Taif 21944, Saudi Arabia; s.abdel-hafez@tu.edu.sa (S.H)
6. Department of Science and Technology, University College-Ranyah, Taif University, Taif 21944, Saudi Arabia; S.sayed@tu.edu.sa (S.SA)

* Correspondence: enas_elsayed@agr.asu.edu.eg (E.H)

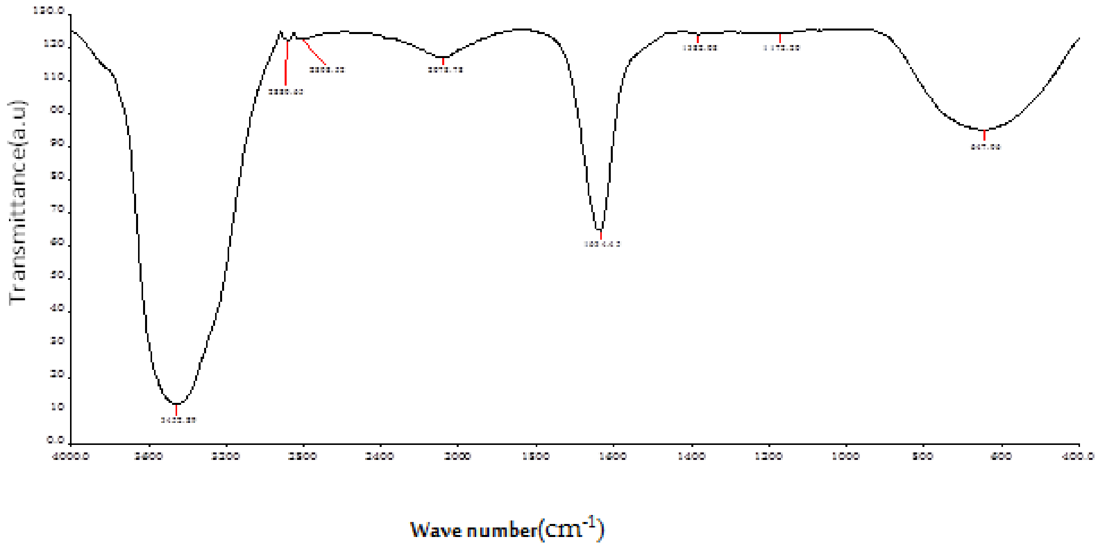

Figure S1. Fourier Transform Infrared spectrum (FTIR) of AgNPs produced by *Streptomyces aizuneusis* ATCC 14921.

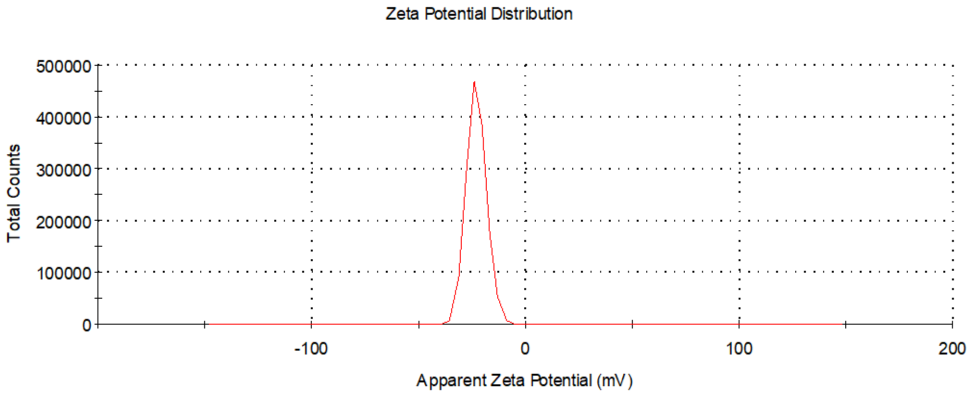

Figure S2. Zeta potential of silver nanoparticles produced by *Streptomyces aizuneusis* ATCC 14921 using zeta sizer analyzer.

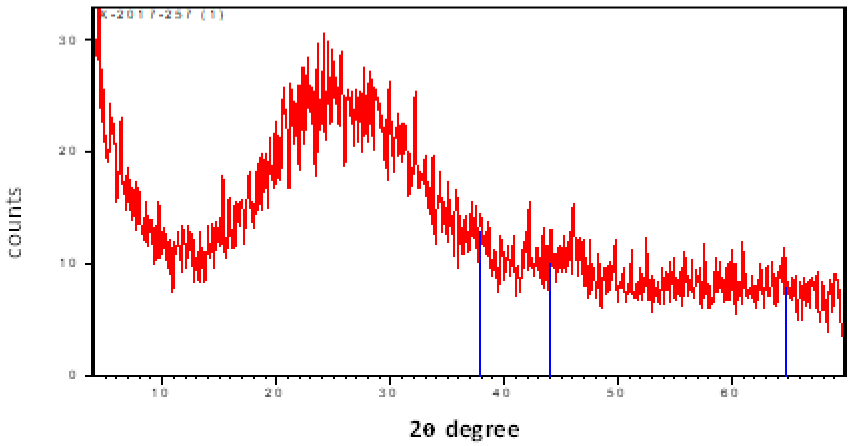

Figure S3. X-ray diffraction of silver nanoparticles produced by *Streptomyces aizuneusis* ATCC 14921. Position (°2θ)(copper (cu)).
